# Neurofilament-Light Chain as Biomarker of Neurodegenerative and Rare Diseases With High Translational Value

**DOI:** 10.3389/fnins.2020.00579

**Published:** 2020-06-11

**Authors:** Tina Loeffler, Irene Schilcher, Stefanie Flunkert, Birgit Hutter-Paier

**Affiliations:** Neuropharmacology, QPS Austria GmbH, Grambach, Austria

**Keywords:** Neurofilament-light chain, biomarker, neurodegenerative disease, lysosomal storage diseases, plasma, cerebrospinal fluid, animal model, preclinical research

## Abstract

Neurofilament-light chain (NF-L) is a well-known clinical biomarker of many neurodegenerative diseases. By analyzing amyotrophic lateral sclerosis (ALS) patients cerebrospinal fluid (CSF) or plasma, progression of NF-L levels can forecast conversion from the presymptomatic to symptomatic stage and time of survival. The use of plasma for NF-L measurement makes this biomarker exceptionally valuable for clinical studies since sample collection can be performed repeatedly without causing much harm. Detailed analyses of NF-L expression in neurodegenerative disease patient’s samples were already performed, while NF-L levels of preclinical models of ALS, Alzheimer’s and Parkinson’s disease as well as lysosomal storage diseases are still widely unknown. We therefore evaluated NF-L levels in the plasma of the ALS models SOD1-G93A low expressor and TAR6/6 mice, the Alzheimer’s disease (AD) model 5xFAD, the Parkinson’s disease model Line 61 and the Gaucher disease (GD) model 4L/PS-NA and the CSF of selected models. Our results show that NF-L levels are highly increased in the plasma of ALS, Alzheimer’s and GD models, while in the analyzed Parkinson’s disease model NF-L plasma levels barely changed. Most analyzed models show a progressive increase of NF-L levels. NF-L measurements in the plasma of the neurodegenerative disease mouse models of ALS and AD are thus a good tool to evaluate disease progression. Compared to analyses in human tissues, our results suggest a high translation value of murine NF-L levels and their progression. Furthermore, our data indicate that NF-L might also be a good biomarker for disorders with a neuronal component like some lysosomal storage diseases.

## Introduction

During the last years, Neurofilament-light chain (NF-L) has been shown to be a valuable biomarker for several neurodegenerative diseases (Gaetani et al., 2019). Since NF-L is located in large caliber myelinated axons of neurons only, most clinical analyses were performed in the cerebrospinal fluid (CSF) of Multiple Sclerosis (MS) patients (Kuhle et al., 2013b) but also tissue of other neurodegenerative diseases were already analyzed in detail. NF-L was thus shown to be strongly increased in the CSF and plasma of Amyotrophic Lateral Sclerosis (ALS) and Alzheimer’s disease (AD) patients (Rosengren et al., 1996; Norgren et al., 2003; Gaiottino et al., 2013; Mattsson et al., 2017). Specifically in ALS, NF-L is shown to be useful as marker for the disease and its progression as it can indicate the conversion from the presymptomatic to symptomatic stage (Tortelli et al., 2012; Benatar et al., 2018). Latest research even suggests that NF-L levels measured in the serum of ALS patients can be used for disease diagnosis and as independent prognostic factor of survival (Thouvenot et al., 2019; Verde et al., 2019). In AD patients, higher NF-L plasma levels correlate with lower Mini-Mental State Examination (MMSE) scores (Mattsson et al., 2017). In patients, pathological NF-L levels were additionally already shown to be reducible by treatment with different compounds (Gunnarsson et al., 2011; Kuhle et al., 2013a; Axelsson et al., 2014; de Flon et al., 2016; Novakova et al., 2017). Although this effect could so far be only presented in MS patients, it provides sufficient evidence, that NF-L could be successfully used as biomarker in clinical studies of neurodegenerative diseases. Due to these results we asked if NF-L might also be a valuable biomarker for preclinical efficacy studies and thus provide an important translational link between preclinical disease models and evaluation of disease progression in patients. A previous study by Bacioglu et al. (2016) already suggests, that NF-L might be increased in PD and AD animals models with α-synuclein, tau and APP mutation. We therefore evaluated the progression of NF-L levels in the plasma of several transgenic animal models of neurodegenerative diseases. Furthermore, we evaluated the NF-L levels in the CSF of selected models to validate the relative expression levels of NF-L in the plasma compared to the CSF as observed in MS patients (Disanto et al., 2017). Zetterberg et al. (2007) could already show an influence of the SOD1 gene on NF-L levels in ALS patients, we therefore decided to evaluate the NF-L levels in SOD1-G93A low expressor mice as preclinical model of ALS (Gurney et al., 1994; Fujisawa et al., 2016). As additional ALS mouse model, also TAR6/6 mice were evaluated (Wils et al., 2010; Scherz et al., 2018) expressing TDP-43, the most widely accumulated protein in ALS patients. As RNA-binding protein, TDP-43 was already shown to interact with NF-L by direct binding to its RNA (Oberstadt et al., 2018). Additionally, we analyzed 5xFAD mice as model of AD (Oakley et al., 2006) and Line 61 mice as model of PD (Rockenstein et al., 2002; Rabl et al., 2017) to evaluate the value of these models in respect of NF-L pathology. Next to these models of classical neurodegenerative diseases we also evaluated NF-L levels in 4L/PS-NA mice as model of Gaucher disease (GD; Sun et al., 2005), a lysosomal storage disease with non-neuronal, and neuronal phenotype. Our study thus shows for the first time plasma NF-L levels in mouse models of PD, AD, ALS, and GD and additional CSF NF-L levels of selected models. Except for GD, all data were evaluated in mice of several age groups providing information about the development of NF-L pathology over age.

## Method

### Animals

All animals were bred and housed under identical conditions in individually ventilated cages on standardized rodent bedding (Rettenmaier^®^, Germany) in the AAALAC-accredited animal facility of QPS-Austria GmbH. Cotton nestlets (Plexx^®^, Netherlands) were provided as nesting material. The room temperature (RT) was kept at approximately 21°C and the relative humidity between 40–70%. Mice were housed under constant light-cycle (12 h light/dark). Dried pelleted standard rodent chow (Altromin^®^, Germany) and normal tap water were available to the animals *ad libitum*. Each individual animal was checked regularly for any clinical sign. Mice were housed in same sex groups of up to four animals. During weaning, animals were marked using ear punch. The punched ear piece taken from each animal was used for genotyping. Age groups vary between mouse models so for each model, a presymptomatic (young), early symptomatic (early adult), and symptomatic (adult) and for some models also late symptomatic (late adult) stage was chosen. For Line 61 no tissue of presymptomatic animals was available. Animals were analyzed cross-sectional. Actual animal numbers are given in the figure legends. Animal studies conformed to the Austrian guidelines for the care and use of laboratory animals (Tierversuchsgesetz 2012-TVG 2012, BGBl. I Nr. 114/2012). Animal housing and euthanasia were approved by the Styrian government (Amt der Steiermärkischen Landesregierung, Abteilung 13–Umwelt und Raumordnung Austria; ABT13-78Jo115/2013-2016; and ABT13-78Jo-118/2013-13).

5xFAD mice (Familiar Alzheimer Disease) bearing 5 mutations, 3 in the APP695 gene [APP K670N/M671L (Swedish), I716V (Florida), V717I (London)] as well as 2 mutations in the presenilin 1 gene (PS1 M146L, L286V) were used (Oakley et al., 2006). The expression of the 5xFAD transgene is driven by the neuron specific Thy-1 promoter. Hemizygous animals of both sexes were bred on a C57BL/6JRccHsd background.

Line 61 mice overexpressing human wild type α-Synuclein under the regulatory control of the Thy-1 promoter with a C57BL/6xDBA background were used (Rockenstein et al., 2002; Rabl et al., 2017). Only hemizygous male animals were used.

TAR6/6 mice expressing human wild type TDP-43 under the control of the murine Thy-1 promoter (Wils et al., 2010; Scherz et al., 2018) on a C57BL/6JRccHsd background were used. Homozygous mice (TAR6/6) of both sexes were bred by mating hemizygous mice (TAR6).

SOD1-G93A low expressor mice [B6.Cg-Tg(SOD1^∗^G93A)^dl^1Gur/J] express a variant of the human superoxide dismutase 1 gene (SOD1) with a glycine to alanine substitution at position 93 (G93A) on a C57BL/6 congenic background. This strain has roughly 30% less copies of the transgene construct than the high copy number line Tg(SOD1^∗^G93A)1Gur (Gurney et al., 1994; Fujisawa et al., 2016). Hemizygous animals of both sexes were purchased from Jackson Laboratories, United States.

Same sex non-transgenic (ntg) littermates of 5xFAD, Line 61, TAR6/6, and SOD1-G93A low expressor mice served as control.

4L/PS-NA mice with homozygous GbaV394L/V394L mutation (4L; Xu et al., 2003), complete prosaposin knockout (PS-), and homozygous prosaposin transgene (NA) resulting in hypomorphic prosaposin levels (Sun et al., 2005), on a mixed FVB/C57Bl/6/129vEvBrd background were bred by mating hemizygous 4L/PS+/-NA mice. Homozygous 4L/PS-NA mice, 4L/PS+/+NA mice with a wild type prosaposin, and C57Bl/6RccHsd mice (Envigo, Italy) of both sexes and of the same age were used.

### Tissue Sampling

All mice were anesthetized by i.p. injection of 600 mg/kg pentobarbital. Once animals were deeply anesthetized, the thorax was opened and blood was drawn by heart puncture of the left ventricle and collected in lithium heparin tubes. Mice were afterwards transcardially perfused with physiological (0.9%) saline. CSF was obtained by blunt dissection and exposure of the foramen magnum. Upon exposure, a Pasteur pipette was inserted at the approximate depth of 0.3–1 mm into the foramen magnum. CSF was collected by suction and capillary action until flow fully ceased. Samples were immediately frozen on dry ice and stored at −80°C until further analysis.

### Plasma Preparation

The blood was centrifuged at 1,000 × *g* for 10 min at RT and plasma transferred to fresh tubes, frozen on dry ice and stored at −80°C until used for analyses.

### Measurement of Neurofilament-Light Chain Levels

For measurement of NF-L levels in terminal plasma samples, the NF-light^®^ ELISA 10-7001 CE (UmanDiagnostics, Sweden) was used without modifications. Specificity of the assay for murine samples was evaluated by Bacioglu and colleagues using NF-L deficient mouse samples as control (Bacioglu et al., 2016). Samples were diluted 1:2.5–1:10 in assay buffer and analyzed using 50 μl per sample according to the manufacturer’s protocol in duplicates on the Cytation 5 multimode reader (Biotek, United States). A minimum dilution of 1:2.5 of plasma and CSF samples was determined upfront to avoid matrix effects. Data were evaluated in comparison to calibration curves provided by the manufacturer and are expressed as pg/mL plasma. All analyzed samples from transgenic animals were well within the detection range of the assay, only some samples from ntg animals were close to or below the LLOQ. Raw data of all analyses are provided in Supplementary Table S1.

### Statistics

Data analysis was performed in GraphPad Prism^TM^ 8.1 (GraphPad Software Inc., United States). Graphs show group means and standard error of the mean (SEM). The significance level was set at *p* < 0.05. Group means were compared using unpaired *t*-test, One-way or Two-way analysis of variance (ANOVA) with a subsequent *post hoc* test. The utilized statistical tests and exact sample numbers are mentioned in the figure legends.

## Results

To evaluate the NF-L levels in different neurodegenerative disease mouse models, NF-L was quantified in Line 61 mice, 5xFAD mice, TAR6/6, and SOD1-G93A low expressor mice as well as 4L/PS-NA mice as model of Parkinson’s disease, AD, ALS, and GD, respectively.

**FIGURE 1:**
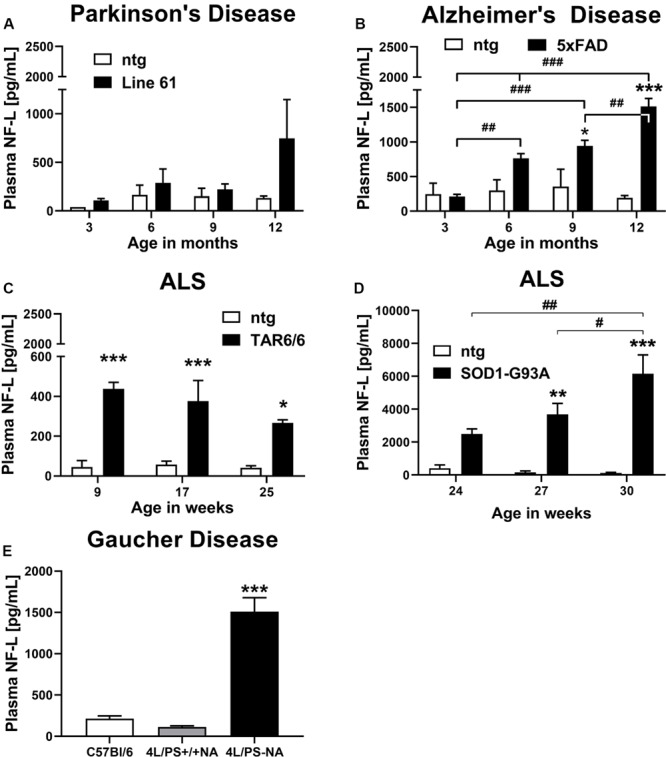
Neurofilament light chain (NF-L) levels in the plasma of different mouse models of neurodegenerative and rare diseases. **(A)** NF-L levels in the plasma of 3 to 12 month old Line 61 mice as model of Parkinson’s disease; Line 61: *n* = 4, ntg: *n* = 2. **(B)** NF-L levels in the plasma of 3 to 12 month old 5xFAD mice as model of Alzheimer’s disease; 5xFAD: *n* = 8, ntg: *n* = 4. **(C)** NF-L levels in the plasma of 9 to 25 week old TAR6/6 mice (TDP-43) as model of Amyotrophic lateral sclerosis; TAR6/6: *n* = 6, ntg: *n* = 5. **(D)** NF-L levels in the plasma of 24 to 30 week old SOD1-G93A low expressor mice as model of Amyotrophic lateral sclerosis; SOD1-G93A: *n* = 6–7, ntg: *n* = 6–7; **(A–D)**: Two-way ANOVA with Bonferroni’s *post hoc* test. *differences between genotypes; #differences between age groups. **(E)** NF-L levels in the plasma of 18 week old 4L/PS-NA mice as model of Gaucher disease; 4L/PS-NA: *n* = 7, 4L/PS+/+NA: *n* = 7, and C57Bl/6: *n* = 7; One-way ANOVA with Tukey’s multiple comparison test. Significance compared to 4L/PS+/+NA littermates and C57Bl/6 mice; **(A–E)**: Mean + SEM. *p < 0.05, **p < 0.01, and ***p < 0.001.

Plasma of male Line 61 mice was analyzed for NF-L levels in 3, 6, 9, and 12 month old animals. The results show that NF-L levels of Line 61 mice might increase in old animals, but statistical analysis revealed no significant differences compared to ntg littermates (Figure 1A). NF-L levels in this Parkinson’s disease mouse model stayed on average below 1,000 pg/mL plasma.

Evaluation of plasma from 3, 6, 9, and 12 month old 5xFAD mice revealed a significant increase of NF-L levels in 9 and 12 month old 5xFAD mice compared to ntg littermates. NF-L levels in 5xFAD mice progressively increased over age (Figure 1B). NF-L levels in this AD mouse model reached on average 1,500 pg/mL plasma in 12 month old animals.

Two mouse models of ALS, TAR6/6, and SOD1-G93A low expressors were analyzed for NF-L levels. In young TDP-43 overexpressing TAR6/6 mice highly increased NF-L levels could be found compared to ntg littermates. This difference slightly decreased in older animals compared to ntg littermates (Figure 1C). Analysis of SOD1-G93A low expressor mice revealed a significant increase of NF-L levels in 27 and 30 week old SOD1-G93A low expressor mice compared to ntg littermates. NF-L levels in SOD1-G93A low expressor mice progressively increased over age (Figure 1D). NF-L levels in the plasma of these ALS mouse models reached on average 400 pg/mL in TAR6/6, and up to 6,000 pg/mL in SOD1-G93A low expressor mice.

Plasma NF-L levels of ntg littermates did not change over age in all analyzed neurodegenerative disease mouse models (Figures 1A–D).

Further analysis of the GD mouse model 4L/PS-NA at an age of 18 weeks revealed highly increased NF-L plasma levels in 4L/PS-NA mice compared to 4L/PS+/+NA and C57Bl/6 mice of the same age. NF-L levels in the plasma of 4L/PS+/+NA and C57Bl/6 mice was not significantly different (Figure 1E). Plasma NF-L levels in 4L/PS-NA mice reached on average 1,500 pg/mL.

**FIGURE 2:**
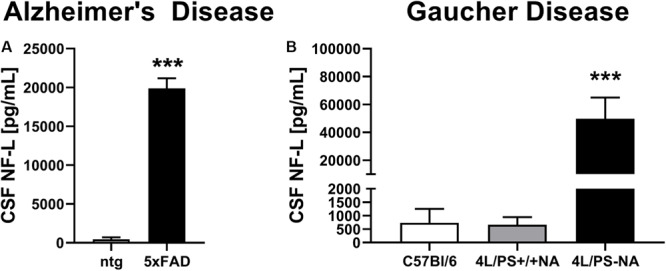
Neurofilament light chain (NF-L) levels in the CSF of Alzheimer’s and Gaucher disease mouse models. **(A)** NF-L levels in the CSF of 9 month old 5xFAD mice as model of Alzheimer’s disease; 5xFAD: *n* = 6, ntg: *n* = 4; unpaired *t*-test; Significance compared to age-matched ntg littermates. **(B)** NF-L levels in the CSF of 18 week old 4L/PS-NA mice as model of Gaucher disease; 4L/PS-NA: *n* = 7, 4L/PS+/+NA: *n* = 7, C57Bl/6: *n* = 4; One-way ANOVA with Tukey’s multiple comparison test. Significance compared to 4L/PS+/+NA littermates and C57Bl/6 mice; **(A,B)**: Mean + SEM; ***p < 0.001.

To validate observed plasma NF-L levels, CSF of the AD mouse model 5xFAD and the GD mouse model 4L/PS-NA was analyzed for NF-L levels. Evaluation of CSF of 9 month old 5xFAD mice revealed significantly increased NF-L levels in 5xFAD mice compared to ntg littermates (Figure 2A). NF-L levels reached up to 20,000 pg/mL and thus approximately 20-fold higher levels compared to the plasma of age-matched 5xFAD mice (Figures 1B, 2A). In 4L/PS-NA mice as model of GD, NF-L levels in the CSF also significantly increased compared to 4L/PS+/+NA and C57Bl/6 mice (Figure 2B). NF-L levels reached up to 50,000 pg/mL in the CSF of 4L/PS-NA mice and thus 30-fold higher values compared to plasma of age-matched 4L/PS-NA mice (Figures 1E, 2B).

## Discussion

Here, we show for the first time the progressive increase of plasma NF-L levels in several mouse models of different neurodegenerative and one rare disease with neuronal component and validate these results by NF-L measurements in the CSF of selected models.

Only the TAR6/6 ALS mouse model does not show a progressive increase in NF-L levels. Although these results are unusual compared to all other analyzed models, it fits to the progression of further neuronal pathologies in TAR6/6 mice. Specifically levels of human TDP protein and glial fibrillary acidic protein (GFAP) were found to be decreased in older TAR6/6 mice (Scherz et al., 2018). The unusual progression of these markers is suggested to be dependent on early neuronal loss of TAR6/6 mice as shown by decreased ChaT-positive neurons in the cervical and lumbar spinal cord (Scherz et al., 2018).

Comparing the here observed progressive increase of NF-L in AD and ALS mouse models with clinical data reviewed by Gaetani et al. (2019) reveals astonishing similarities. In both cases are the NF-L levels much higher in ALS compared to AD and PD. In ALS patients CSF NF-L levels were about 20-fold higher compared to healthy controls. Here we found plasma NF-L levels to be up to 46-fold increased compared to ntg littermates. In AD patients CSF NF-L levels were about 2-fold higher compared to healthy controls (Gaetani et al., 2019). Here we found NF-L levels to be 45-fold higher in the CFS of 5xFAD mice while plasma levels were up to 7-fold increased compared to ntg littermates. The analyzed ALS and AD animal models thus present a much stronger NF-L pathology compared to evaluated patient tissue but the overall trend between different neurodegenerative diseases stays the same.

Analysis of plasma of the PD mouse model Line 61 that is known to present a strong and solid behavioral and pathological phenotype (Chesselet et al., 2012; Rabl et al., 2017) showed only a minor increase of NF-L levels with a strong variation in older animals. These results also compare well with PD patients who show almost no increase in NF-L levels making PD the neurodegenerative disease with the weakest NF-L pathology (Gaetani et al., 2019).

Next to the evaluation of NF-L in different neurodegenerative disease mouse models we also analyzed NF-L levels in the CSF and plasma of the GD mouse model 4L/PS-NA observing a 9-fold increase in the plasma and a 70-fold increase in the CSF compared to controls. These data suggest, that the NF-L pathology of 4L/PS-NA mice is stronger compared to 5xFAD mice but weaker compared to SOD1-low expressor mice, placing the GD NF-L levels pathologically between AD and ALS.

Furthermore, observed changes in NF-L levels of GD mice suggest that NF-L might not only be a valuable biomarker in classical neurodegenerative diseases but also in other diseases that have a neuronal component like some lysosomal storage diseases, e.g., GD, Pompe disease, Krabbe disease, or Sanfilippo syndrome. For patients of the lysosomal storage disease Niemann-Pick a first study that confirms the value of NF-L as CSF marker was just released (Eratne et al., 2019). Analysis of GD patients CSF and/or plasma will hence reveal, if NF-L could have a similar value for the measurement of disease progression as observed for ALS patients.

As increased NF-L levels in the CSF and plasma are suggested to be indicative for an increased loss of neurons that possess a large dendritic tree or even more importantly larger myelinated axons our results suggest the strongest neuronal loss in ALS, followed by GD, AD and the least in PD mouse models. Motor neuron death in SOD1-G93A low expressor mice was already previously shown for the spinal cord (Fujisawa et al., 2016). One of the first publications about neuronal death in the high expressor SOD1 mouse model [(SOD1^∗^G93A)1Gur/J] that is used by most ALS researchers also analyzed neurofilament proteins in the lumbar spinal cord and found reduced neurofilament reactivity caused by a loss of motor neurons and axons (Feeney et al., 2001). These results already provided the first hint for altered neurofilament protein levels and aggregation in high expressor SOD1 mice and thus laid the foundation for future neurofilament research in ALS. Neuronal loss in TAR6/6 mice was already described above to be shown by the reduction of ChaT-positive cells in the spinal cord (Scherz et al., 2018) and was additionally shown in the motor cortex layer V of 6 month old TAR6/6 mice (Wils et al., 2010). All results about neuronal death in SOD1 and TAR6/6 mouse models thus support the here observed strong NF-L levels in these mice to be indicative for strong neuronal loss.

Recent results show a strong Purkinje cell loss in the cerebellar network of 4L/PS-NA mice (Schiffer et al., 2020) and thus validating previous findings that link Purkinje cell loss to saposin D deficiency (Matsuda et al., 2004) as it is caused by reduced prosapsin expression in 4L/PS-NA mice (Sun et al., 2005). Future studies will have to show, if the observed increase in plasma and CSF NF-L levels in 4L/PS-NA mice is solely initiated by the cerebellar neuronal loss or if also other brain regions are affected.

5xFAD transgenic mice are well-described to present neuronal loss in cortical layer 5 and in the subiculum at 9 month of age (Eimer and Vassar, 2013) and in the hippocampus already at the age of 7 month (Modi et al., 2014). These results reflect very well the here observed strong NF-L pathology in this AD mouse model.

Contrary, in Line 61, the model with the least NF-L pathology, only one publication points toward an apoptotic signature in the striatum of Line 61 mice as analyzed by transcriptome analysis that is paralleled by the activation of an adaptive compensatory mechanism probably protecting striatal neurons from cell death (Cabeza-Arvelaiz et al., 2011). The here observed NF-L levels thus fit well to previous findings that could not confirm neuronal death in Line 61 mice.

In familial AD, NF-L levels are shown to increase already at a presymptomatic stage, supporting its value as clinical biomarker (Preische et al., 2019). In contrast, in 5xFAD mice NF-L levels in presymptomatic animals were as low as in control mice, allowing the use of presymptomatic 5xFAD as baseline for NF-L measurements in preclinical studies. In SOD1 low expressor mice, also presymptomatic animals already showed a clear trend toward increased NF-L levels that would prevent the use of tissue from presymptomatic SOD1 low expressor mice as baseline for NF-L measurement but reflects the results observed in human AD tissue. For the evaluation of NF-L levels in TAR6/6 mice, only animals of an early symptomatic stage were available. At this age, NF-L levels were already highly increased, suggesting that probably also presymptomatic TAR6/6 mice present increased NF-L levels. Further evaluations are needed to clearly define the onset of increased plasma NF-L levels in TAR6/6 mice. NF-L measurement in preclinical animal models should thus provide a valuable tool to measure the degree of neuronal death in efficacy studies to test new compounds against neurodegenerative and related diseases. This read-out should additionally be easily used for longitudinal studies by using repeated blood sampling that is less invasive for animals compared to repeated CSF sampling.

## Conclusion

Evaluation of NF-L plasma levels in various neurodegenerative disease mouse models showed similar changes compared to already published corresponding patients tissue, suggesting that these mouse models have a high translational value for preclinical research studies. Due to the option of repeated blood sampling followed by NF-L measurement in the plasma, this method could decrease the number of animals needed for the analysis of neuronal cell death in preclinical efficacy studies. Furthermore our results suggest that NF-L might also be a suitable biomarker for diseases that are not classical neurodegenerative diseases but contain a neuronal component like some lysosomal storage diseases.

## Data Availability Statement

All datasets generated for this study are included in the article/Supplementary Material.

## Ethics Statement

The animal study was reviewed and approved by Amt der Steiermärkischen Landesregierung Abteilung 13–Umwelt und Raumordnung Stempfergasse 7 8010 Graz.

## Author Contributions

TL and IS designed, performed and analyzed all experiments, and edited the manuscript. SF prepared figures, interpreted results, and wrote manuscript. BH-P conceived the study, interpreted results, and edited the manuscript.

## Conflict of Interest

All authors are employees of QPS Austria GmbH.

## Supplementary Material

TABLE S1 Raw data of Figures 1, 2.

## Abbreviations

AAALAC: Association for Assessment and Accreditation of Laboratory Animal Care
AD: Alzheimer’s Disease
ALS: Amyotrophic Lateral Sclerosis
ANOVA: Analysis of variance
APP: Amyloid Precursor Protein
CNS: central nervous system
CSF: cerebrospinal fluid
FAD: familial Alzheimer’s disease
GD: Gaucher Disease
GFAP: glial fibrillary acidic protein
LLOQ: lower limit of quantification
MMSE: Mini-Mental State Examination
MS: Multiple Sclerosis
NF-L: Neurofilament light-chain
ntg: non-transgenic
PD: Parkinson’s Disease
SEM: Standard error mean
SOD1: superoxide dismutase 1 gene
TDP-43: TAR DNA-binding protein 43.
